# Can Loneliness be Predicted? Development of a Risk Prediction Model for Loneliness among Elderly Chinese: A Study Based on CLHLS

**DOI:** 10.21203/rs.3.rs-4773143/v1

**Published:** 2024-09-02

**Authors:** Youbei Lin, Chuang Li, Hongyu Li, Xiuli Wang

**Affiliations:** The First Affiliated Hospital of Jinzhou Medical University; Jinzhou Medical University; Jinzhou Medical University; The First Affiliated Hospital of Jinzhou Medical University

**Keywords:** Older adults, Loneliness, Predictive Models, Machine Learning

## Abstract

**Background:**

Loneliness is prevalent among the elderly, worsened by global aging trends. It impacts mental and physiological health. Traditional scales for measuring loneliness may be biased due to cognitive decline and varying definitions. Machine learning advancements offer potential improvements in risk prediction models.

**Methods:**

Data from the 2018 Chinese Longitudinal Healthy Longevity Survey (CLHLS), involving over 16,000 participants aged ≥65 years, were used. The study examined the relationships between loneliness and factors such as cognitive function, functional limitations, living conditions, environmental influences, age-related health issues, and health behaviors. Using R 4.4.1, seven predictive models were developed: logistic regression, ridge regression, support vector machines, K-nearest neighbors, decision trees, random forests, and multi-layer perceptron. Models were evaluated based on ROC curves, accuracy, precision, recall, F1 scores, and AUC.

**Results:**

Loneliness prevalence among elderly Chinese was 23.4%. Analysis identified 16 predictive factors and evaluated seven models. Logistic regression was the most effective model for predicting loneliness risk due to its economic and operational advantages.

**Conclusion:**

The study found a 23.4% prevalence of loneliness among elderly individuals in China. SHAP values indicated that higher MMSE scores correlate with lower loneliness levels. Logistic regression was the superior model for predicting loneliness risk in this population.

## 1. Introduction

According to recent reports from the World Health Organization, approximately 25% of elderly individuals worldwide experience feelings of loneliness.(Teo et al., 2023) However, the concept of loneliness remains elusive. Weiss, a pioneer in loneliness research, introduced the Emotional Support Model, which posits loneliness as an emotional state reflecting deficiencies in social relationships. Specifically, Weiss delineated two types of loneliness: social loneliness and emotional loneliness(Russell et al., 1984). Social loneliness arises from a lack of broad social contacts and interactions, reflecting a need for a wider social network. Emotional loneliness stems from a deficit in deep emotional support and intimate relationships, reflecting a need for significant emotional connections.(Chistell et al., 2023) Weiss emphasizes that understanding loneliness necessitates analyzing these dimensions to identify deficiencies in social relationships.(Weiss, 1975)This model helps explain why some individuals may feel lonely despite having many friends, potentially due to lacking genuine emotional connections. Conversely, others with few friends may not feel lonely if they have close intimate relationships. The framework provided by Weiss enables researchers and practitioners to comprehensively understand and address loneliness, facilitating targeted interventions to meet individuals’ social and emotional needs. Additionally, the concept of social isolation, akin to loneliness, remains undefined. Social isolation typically refers to a state characterized by reduced social interactions and a diminished social circle, measured objectively through indicators such as living alone, marital status, and social frequency.(Lai et al., 2024) Despite conceptual distinctions, social isolation interacts with loneliness. Chinese scholar Wang Xue applied Rodgers’ evolutionary concept analysis to explore loneliness among the elderly, defining its attributes as deficits in intimate relationships, social isolation, and self-alienation, highlighting how social isolation can contribute to loneliness.(Wang et al., 2023) Current studies predominantly focus on interventions targeting loneliness among the elderly, as well as exploring individual differences in factors related to loneliness across elderly populations. Another research direction examines the impact of loneliness on physiological illnesses.

In recent years, machine learning-based risk prediction models have emerged in the field of emotional and affective domains. (Richter et al., 2021)Thus, developing a risk prediction model for loneliness among elderly individuals based on machine learning represents a promising endeavor. Factors influencing loneliness among the elderly are still under exploration, encompassing demographic and social factors, physiological conditions, psychological factors, social and living environments. Demographic and social factors include age, gender, marital status, and education level. Increasing age often accompanies transitions in social roles, such as retirement and children leaving home, potentially reducing social opportunities. Gender differences manifest significantly in experiences of loneliness; for instance, women may be more susceptible to loneliness following widowhood.(Lim et al., 2022) Marital status significantly influences loneliness; married elderly individuals generally experience less loneliness than unmarried, divorced, or widowed individuals. (Koren et al., 2024)Education level may impact the breadth and quality of social networks, with higher-educated elderly individuals typically possessing more social resources.(Sanchez et al., 2024) Physiological factors encompass health status, chronic illnesses, and physical impairments. Poor health among the elderly may reduce social activities due to decreased physical ability, exacerbating loneliness.(Sunwoo, 2020) Chronic illnesses such as heart disease and diabetes not only affect physical health but also negatively impact mental health. Physical impairments, such as hearing or vision impairments, may hinder communication with the outside world, further exacerbating loneliness.(Thompson et al., 2024) Psychological factors such as depression, anxiety, and self-esteem directly influence feelings of loneliness. Depression symptoms often co-occur with loneliness and may mutually reinforce each other.(Liang et al., 2023) Anxiety may reduce social interactions among the elderly, increasing loneliness. Additionally, elderly individuals with low self-esteem may perceive social rejection more readily, contributing to loneliness.(Perlman & Peplau, 1981) Cognitive decline, including memory loss and cognitive impairment, may limit social capabilities and opportunities, thereby increasing loneliness. (Camacho et al., 2024)Social environment involves social support networks and social participation levels. Strong social support networks, such as close family relationships and friendships, effectively alleviate loneliness. (Hogan et al., 2002)Social participation levels, such as engagement in community activities, volunteering, or social gatherings, negatively correlate with loneliness. (Zhang et al., 2018)Social support and participation not only provide emotional support but also alleviate loneliness through practical assistance. Living environment factors include housing conditions, community safety, and neighbor relationships. Good housing conditions, such as comfortable living environments and convenient community facilities, improve elderly individuals’ quality of life and reduce loneliness. Community safety encourages elderly individuals to engage in social activities. Positive neighbor relationships provide daily social interactions and emotional support, reducing loneliness.(Bhuyan & Yuen, 2022)

Existing studies have extensively explored the causes and impacts of loneliness through methods such as questionnaires, interviews, and psychological assessments. These studies offer valuable insights into the determinants of loneliness and its effects on elderly populations. However, most studies primarily conduct post-hoc analyses to identify the presence and severity of loneliness, limiting the ability to provide timely and effective intervention strategies. Proactive research on prospective predictions of loneliness risk among the elderly is noticeably lacking in the literature. Accurate prediction models can assist healthcare providers, policymakers, and caregivers in identifying high-risk individuals before loneliness becomes a severe issue, thereby promoting early intervention to potentially mitigate the negative impacts of loneliness.

The primary aim of this study is to develop a machine learning-based predictive model to assess loneliness risk among elderly individuals. This model aims to comprehensively consider various factors, including demographic and social factors, physiological factors, psychological factors, social environment, and living environment, to provide a comprehensive risk assessment. By accurately predicting loneliness risk, the model aims to guide the development of targeted intervention strategies to improve the quality of life for elderly individuals. In this paper, we first review existing literature on loneliness among the elderly, highlighting the strengths and limitations of current methodologies. This review provides background for understanding the complexity of loneliness and the necessity for predictive models. Following the literature review, we detail our research methodology, including the dataset used, variable selection process, and model construction techniques. The dataset comprises multiple variables assumed to influence loneliness, selected based on theoretical considerations and empirical evidence. We constructed seven machine learning models, including logistic regression, ridge regression, support vector machines, decision trees, random forests, K-nearest neighbors algorithm, and multilayer perceptron. The model construction section outlines the statistical and computational techniques for each model. We employed cross-validation methods to evaluate model performance and used multiple evaluation metrics (such as accuracy, recall, F1 score, etc.) for comprehensive model comparison. Through detailed performance evaluation, we identified the optimal model, its practical application value, and potential applications. Finally, we discuss the limitations of the study and propose future research directions. The limitations section acknowledges challenges encountered in data availability and the generalizability of research findings. Despite these limitations, this study contributes to the existing knowledge base by offering a new approach to predicting loneliness risk among elderly individuals. By identifying high-risk individuals early, this model has the potential to inform targeted intervention strategies, thereby improving elderly well-being. The study underscores the importance of adopting proactive approaches in addressing loneliness and contributes to broader efforts aimed at enhancing the quality of life for elderly individuals.

## 2. Methodology

### 2.1 Study Design and Participants

The Chinese Longitudinal Healthy Longevity Survey (CLHLS) is an elderly tracking survey organized by the Center for Healthy Aging and Development Studies at Peking University/National Development Research Institute. It covers 23 provinces, municipalities, and autonomous regions nationwide, targeting individuals aged 65 and older as well as their adult children aged 35–64. The survey consists of two types of questionnaires: one for living participants and another for deceased participants’ family members. The living participant questionnaire covers basic elderly and family conditions, socio-economic background, family structure, economic sources and status, self-rated health and quality of life, cognitive function, personality traits, daily activity ability, lifestyle, caregiving, disease treatment, and medical expenses burden. The questionnaire for deceased participants’ family members collects information on the time and cause of death. The survey commenced with a baseline study in 1998 and subsequent follow-ups were conducted in 2000, 2002, 2005, 2008–2009, 2011–2012, 2014, and 2017–2018. The most recent follow-up in 2017–2018 included 15,874 elderly participants aged 65 and older, collecting information on 2,226 deceased elderly individuals between 2014 and 2018.(Center for Healthy & Development, 2020)

For this study, we utilized the cross-sectional database from 2018, comprising approximately 16,000 elderly individuals aged 65 and above. During the data processing stage, selected questions were recoded and corresponding scale scores were computed. Loneliness was assessed by the question “Do you often feel lonely?” with responses categorized into “always,” “often,” “sometimes,” “rarely,” and “never,” as validated in previous research for loneliness assessment. For statistical analysis, responses were recoded into a binary variable: “always,” “often,” and “sometimes” were defined as “feeling lonely (FL, 23.4%),” while “rarely” and “never” were defined as “not feeling lonely (NFL, 76.6%).” (Wei et al., 2022)Functional limitations were assessed using the Katz Activities of Daily Living (ADL) scale and the Lawton Instrumental Activities of Daily Living (IADL) scale. Difficulty performing any of the ADL tasks (bathing, dressing, toileting, transferring, continence, eating) or IADL tasks (visiting neighbors, shopping, cooking, doing laundry, walking 1 km, carrying 5 kg weight, kneeling and standing 3 times, using public transportation) was defined as having ADL or IADL limitations, respectively.(Wei et al., 2022) Covariates included baseline demographic measurements: gender (0 = female/1 = male), marital status (1 = married/2 = separated but married/3 = divorced/4 = widowed/5 = single), years of education, adequacy of life sources (0 = insufficient/1 = sufficient), satisfaction with current life (0 = dissatisfied/1 = satisfied), satisfaction with health status (0 = dissatisfied/1 = satisfied), elderly personality (0 = introverted/1 = extroverted), living conditions of elderly respondents (0 = urban/1 = rural), current living arrangements (1 = with family/2 = living alone/3 = in care institutions), environmental factors (0 = no musty odor at home/1 = yes), use of air purification devices or activated carbon to improve indoor air quality at home (0 = no/1 = yes), age-related health issues: visual impairment (0 = unimpaired/1 = impaired), hearing impairment (0 = unimpaired/1 = impaired), toothache, cheek or jaw pain in the past six months (0 = no/1 = yes), history of falls in the past year (0 = no/1 = yes), and chronic diseases (summarized as the number of chronic diseases). Personal health behaviors included current smoking status (0 = no/1 = yes), regular alcohol consumption (0 = no/1 = yes), regular physical exercise (0 = no/1 = yes), regular calcium supplement intake (0 = no/1 = yes), and social engagement: responses were recoded into a binary variable: “almost every day,” “at least once a week but not daily,” “at least once a month but not weekly,” “sometimes” were defined as participating in social activities, while “never” was defined as not participating in social activities. Data cleaning involved imputation for missing data: continuous variables (actual age, MMSE score, Katz ADL score, Lawton IADL score, years of education) were imputed with mean values, and unordered discrete variables such as questions in covariates were imputed with mode values. Data for participants lost to follow-up were excluded from analysis.

### 2.2 Statistical Analysis

Construction of a Risk Prediction Model for Loneliness Among Chinese Elderly:

#### 2.2.1 Feature Engineering:

In the feature selection stage, this study employed a combination of univariate and multivariate analyses to select variables from the aforementioned set, focusing on variables with P ≤ 0.001. These variables were collected for the subsequent construction of the loneliness risk prediction model for the elderly. (Table 1: Summary of Variable Feature Selection: Integration of Univariate and Multivariate Analysis)

#### 2.2.2 Data Standardization:

To enhance feature interpretability and standardize variable units, Min-Max normalization was employed in this study. This normalization method ensures that all non-binary variables are uniformly scaled. After Min-Max normalization, the importance of features in regression-based machine learning models (such as logistic regression) becomes easily interpretable.(Cabello-Solorzano et al., 2023)

#### 2.2.3 Data Splitting:

The study utilized R version 4.4.1 code to split data into training and validation sets at a ratio of 7:3.

#### 2.2.4 Model Selection:

This study considered several machine learning models: Logistic Regression, Ridge Regression (Ridge C), Linear Support Vector Machine (SVM-Linear), K-Nearest Neighbors (KNN), Decision Tree (DT), Random Forest (RF), and Multi-Layer Perceptron (MLP). Logistic Regression is widely used in medicine for analyzing and predicting binary outcomes due to its strong ability to handle binary classification problems, interpret model coefficients, provide probability outputs, and its simplicity in computation and implementation.(Hassan et al., 2021) Ridge Regression introduces a penalty term in the loss function to constrain the size of regression coefficients, reducing model complexity and overfitting risks, making it suitable for medical statistical analysis and decision support.(van Wieringen, 2015) SVM is a popular machine learning algorithm used for classification and regression analyses, with SVM-Linear specifically effective in handling linearly separable data, high-dimensional data, and performing well on small sample datasets in medical research and practice.(Salcedo-Sanz et al., 2014) KNN is a simple and widely used supervised learning algorithm for classification and regression, based on measuring distances between new samples and training samples to predict outcomes based on the nearest neighbors’ categories or values.(Taunk et al., 2019) DT is a common supervised learning algorithm applicable to classification and regression problems, using a hierarchical approach to partition data into subsets, forming a tree structure where nodes represent features and edges represent feature values, with leaf nodes indicating final classification or prediction results.(Maimon & Rokach, 2014) RF is an ensemble learning method constructing multiple decision trees and combining their results for classification and regression, enhancing model accuracy, robustness, and handling of high-dimensional data and missing values, thus effective in medical research and practice.(Qi, 2012) MLP is a feedforward neural network composed of an input layer, one or more hidden layers, and an output layer, using weighted connections and activation functions for non-linear transformations.(Sharma et al., 2017) MLP finds broad applications in medicine for disease diagnosis (e.g., cardiovascular disease(Deepika & Balaji, 2022) and diabetes classification(Theerthagiri et al., 2022)), medical image analysis(Jiang et al., 2010) (detection and classification of lesions), bioinformatics(Xie et al., 2017) (analysis of gene expression data), and personalized medicine(Norgeot, 2019) (providing personalized treatment recommendations). MLP excels in handling complex non-linear relationships, strong fitting capabilities, and versatility but requires careful tuning of hyperparameters, longer training times, and substantial data to avoid overfitting.(Wazirali et al., 2023)

#### 2.2.5 Model Training and Validation:

The loneliness risk prediction model for the elderly was constructed using R, incorporating K-fold cross-validation to ensure model stability.(Chen et al., 2024)

#### 2.2.6 Model Evaluation:

The constructed machine learning models were evaluated using metrics such as ROC-AUC, precision, accuracy, recall, and F1 score(Alghamdi et al., 2020) to comprehensively assess and identify the optimal risk prediction model for loneliness among Chinese elderly.

## 3. Results

### 3.1 Loneliness

The occurrence rates of FL (Feeling Lonely) and NFL (Not Feeling Lonely) among the elderly were 23.4% and 76.6%, respectively. Table 1 presents the results of the feature selection of variables adopted in this study. A total of 15 variables were included, namely Katz scale, IADL, number of chronic diseases, current living arrangements, presence of mildew odor in the home, self-reported quality of life, self-reported health, optimistic outlook, current drinking status, current exercise status, social interaction with friends, sufficiency of financial support for daily expenses, current marital status, visual function regarding perceiving a break in a circle, and calcium supplement intake.

### 3.2 Development and Evaluation of Risk Prediction Models

In this study, we utilized data from 15,874 individuals to construct and evaluate risk prediction models for loneliness among the elderly. The data were randomly divided into training and validation sets at a ratio of 7:3, with 11,112 cases included in the training set and the remaining 4,762 cases in the validation set. Homogeneity of the training and validation sets was verified using R language, aiming to reduce statistical bias and ensure results closer to real values (see Table 2). During the initial model construction phase, all 16 predictor variables were used as input, with loneliness (FL) as the output variable. Using the training set, we employed the glmnet, svmLinear, knn, rpart, rf, and nnet packages in R 4.4.1 to build seven machine learning models: Logistic Regression, Ridge Regression, SVM-Linear, K-Nearest Neighbors (KNN), Decision Tree (DT), Random Forest (RF), and Multi-Layer Perceptron (MLP). K-fold cross-validation was employed to ensure model stability.

The homogeneity test between the training and validation sets was conducted to minimize statistical bias and ensure that the results more accurately reflect true values.

**Figure 2:**
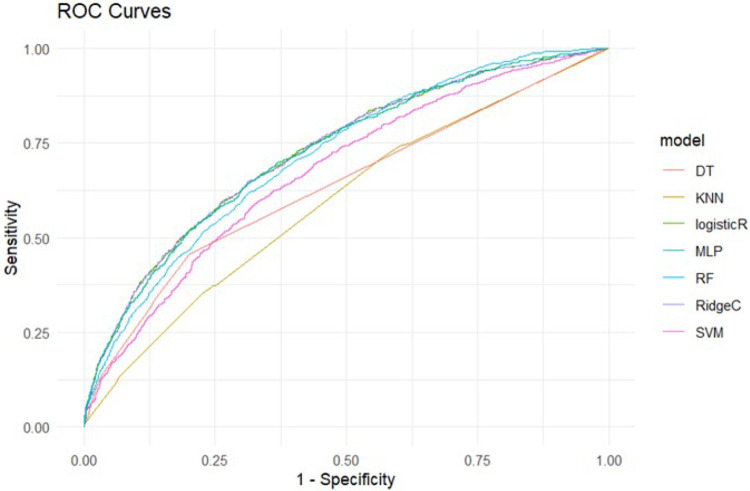
ROC Curve Summary for Seven Models

To assess the predictive performance of the constructed models, we used the validation set data and plotted Receiver Operating Characteristic (ROC) curves, calculating the Area Under the Curve (AUC) values. The ROC curve is a graphical method that displays model performance by changing the discrimination threshold, while the AUC represents the area under the ROC curve, ranging from 0.5 (random prediction) to 1 (perfect prediction). An AUC of 1.0 indicates a perfect test, whereas in general, an AUC of 0.9–0.99 is an excellent test, 0.8–0.89 a good test, 0.7–0.79 a fair test, and < 0.7 a nonuseful test.(Carter et al., 2016) Accuracy is an intuitive and easily understood metric but may be misleading in cases of imbalanced sample classes (where positive and negative samples differ greatly in number).(Wang et al., 2021) For instance, if a disease is very rare in the overall population, a model that simply predicts everyone as non-diseased could achieve high accuracy but would be of little practical use in identifying actual disease cases. To address this issue, additional evaluation metrics such as Precision, Recall, and F1 Score are typically combined to comprehensively assess model performance.(Schrodi et al., 2014) Precision, also known as Positive Predictive Value (PPV), is a metric for evaluating model performance in classification problems.(Tharwat, 2021) It measures the proportion of predicted positive class samples that are actually positive. In other words, precision evaluates the accuracy of predictions made by the model. F1 Score is the harmonic mean of precision and recall, used to provide a balanced assessment of model performance.(Xu et al., 2022) Fig. 1 presents the comprehensive evaluation results of seven machine learning models used in this study for predicting loneliness among the elderly. The evaluation metrics used include ROC curves with corresponding AUC values, precision, recall, and F1 scores. These metrics collectively assess the predictive performance of each model based on the validation dataset. The figure provides a comparative analysis of how each model performed across these metrics, highlighting strengths and potential areas for improvement in their predictive capabilities for loneliness among older adults. Figure 2: Summary of ROC Curves for Seven Models.

This figure presents the evaluation metrics used for assessing the performance of seven risk prediction models constructed using machine learning techniques.

This figure displays the combined ROC curves for the seven risk prediction models. The models include: DT (Decision Tree), KNN (k-Nearest Neighbors), Logistic R (Logistic Regression), MLP (Multilayer Perceptron), RF (Random Forest), RidgeC (Ridge Regression), and SVM (Support Vector Machine).

### 3.3 Interpretability Analysis Based on the SHAP Algorithm

**Figure 3:**
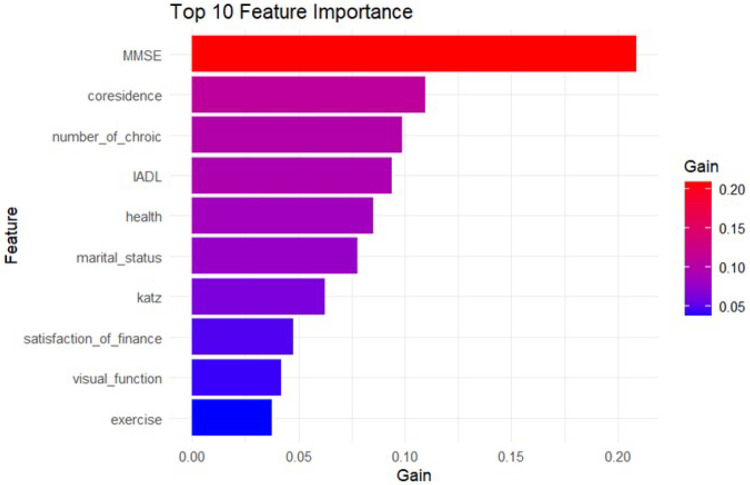
Presents the SHAP feature importance ranking

SHAP is a visualization method based on game theory used to interpret machine learning model outputs.(Petch et al., 2022) Some researchers have successfully employed this algorithm to overcome the “black box” nature of machine learning, providing consistent interpretability for models. Studies(Fainberg et al., 2024) have shown that interpretable machine learning offers new insights for explaining complex and heterogeneous biological data. This study employs Python 3.12 and PyCharm software to compute and visualize SHAP values, using the SHAP algorithm to provide interpretability analysis for the MLP model and visualize all features. The SHAP feature importance ranking indicates that marital status has the strongest predictive value across all forecasting periods. Specifically, elderly individuals who are never married, widowed, divorced, or separated are more likely to experience loneliness compared to their married counterparts. Additionally, features such as cohabitation status and self-rated health of the elderly also hold significant predictive value. (Fig. 3 SHAP summary plot.) Prior to this visualization, the study utilized the Pairplot function in Python to examine the relationships and data distribution characteristics between loneliness and independent variables, and to display the most significant features. The top five features depicted are the Kazt scale, IADL scale, number of chronic diseases, living alone, and residing in care facilities. (Fig. 4 the Pairplot of the top five important features.)

This figure illustrates the SHAP summary plot, which provides an overview of feature importance in the model. This plot visualizes the impact of each feature on the model’s predictions, showing the relative importance of features such as marital status, co-residence, and self-rated health. The summary plot helps in understanding how different features contribute to the prediction of loneliness among the elderly.

Figure 4 The top five important features

This figure presents the Pairplot visualization of the top five important features identified in the analysis. This plot illustrates the relationships and distributions of the Kazt scale, IADL scale, number of chronic diseases, living alone, and residing in care facilities. The Pairplot effectively highlights the correlations and patterns among these features with respect to loneliness in the elderly population.

## 4. Discussion

This study developed a predictive model based on machine learning algorithms to assess the risk of loneliness among elderly individuals in China. By integrating various demographic, physiological, psychological, social, and environmental variables, we constructed and compared seven machine learning models: logistic regression, ridge regression, support vector machine, random forest, decision tree, k-nearest neighbors, and multi-layer perceptron (MLP). Our findings indicate that logistic regression and MLP performed best in predicting loneliness risk among the elderly, demonstrating high accuracy and recall rates. Logistic regression excelled due to its simplicity and interpretability, particularly advantageous for handling high-dimensional data and multicollinearity issues. MLP, as a neural network model, effectively captured complex non-linear relationships within the data, demonstrating outstanding performance in complex loneliness prediction tasks.

To identify significant features associated with loneliness among elderly individuals in China, this study employs the SHAP algorithm to interpret a logistic regression model. The SHAP algorithm estimates feature importance by assigning Shapley values, which reflect the optimal contribution of each feature. (Bifarin, 2023) In the model, the five features with the most substantial impact on elderly loneliness are: marital status, cohabitation status, self-rated health (a protective factor), frequency of social interactions with friends, and functional limitations. Pairplot visualization of these key features reveals that functional limitations (as measured by the Katz and IADL scales) and lack of social support (living alone and residing in care facilities) are prominent. These findings provide additional support for Weiss’s theory of loneliness, which differentiates between emotional and social loneliness.

This study highlights a significant relationship between loneliness and marital status. Chaya’s phenomenological research on elderly individuals who are divorced in later life explores their experiences of freedom and loneliness from a dual-family perspective through semi-structured interviews. The study identifies generational gaps regarding the benefits and costs of late-life divorce. While most elderly individuals view late-life divorce as emphasizing the benefits of freedom, their adult children often describe the drawbacks of loneliness, perceiving both loneliness and freedom as negative aspects.(Koren et al., 2024) For single elderly individuals, their experience of loneliness is closely linked to their social support networks.(Golden et al., 2009) The absence of a spouse often results in a smaller social circle and less frequent interaction with friends and family, thereby increasing social loneliness. (Teater et al., 2021)Additionally, single elderly individuals may experience lower life satisfaction due to a lack of intimate relationships, exacerbating emotional loneliness. (Park et al., 2021) Widowhood entails the loss of long-term companionship and support, which can lead to profound feelings of loneliness and grief.(King et al., 2021) The death of a partner often results in feelings of isolation, especially in the absence of other close relationships.(Pietromonaco & Overall, 2022) Widowhood itself acts as a stressor, requiring elderly individuals to cope with the emotional trauma of losing a partner while adapting to a new life phase. (Carr, 2020) Reduced physical and cognitive functions in the elderly further hinder their ability to adjust effectively. Interventions for single, divorced, separated, and widowed elderly individuals typically focus on enhancing emotional and social support to enrich their internal well-being. With advancements in artificial intelligence, robots and generative AI are emerging as potential tools for emotional support. Bahar Irfan(Irfan et al., 2024) has provided actionable recommendations for designing conversational companion robots for the elderly, utilizing foundational models such as LLMs and visual-language models. These models aim to offer social and emotional support to alleviate loneliness and social isolation in elderly individuals. Chiang Liang Kok (Kok et al., 2024)has developed a social robot designed to bridge the gap between humans and machines, integrating embedded systems, robotics, and basic soft skills to enable effective interactions. This technological solution is anticipated to address caregiver shortages, reduce elderly individuals’ feelings of isolation, and potentially transform elderly care through innovative applications, thereby improving their overall well-being.

Despite these significant findings, several limitations should be acknowledged. Firstly, the data predominantly originated from China, necessitating further validation of model accuracy among elderly populations with diverse cultural backgrounds and living environments. Future research should consider cross-cultural and cross-regional comparative studies to validate model generalizability and stability. Secondly, our study relied on cross-sectional data, limiting insights into the dynamic changes and causal relationships of loneliness. Future studies should adopt longitudinal designs to track changes in loneliness among the elderly, providing deeper insights into its mechanisms and causal relationships. Furthermore, while our predictive models demonstrated high accuracy, further optimization and refinement are warranted. For instance, integrating additional biomarkers and behavioral data may enhance predictive capabilities. Additionally, other factors closely related to elderly living, such as socioeconomic status, cultural background, and social networks, should be considered to construct a more comprehensive and accurate loneliness prediction model. Lastly, although various machine learning models were employed in this study, their complexity and computational costs may restrict practical applications. Future research should explore early intervention strategies for loneliness, particularly personalized interventions for high-risk elderly individuals. Community interventions, psychological counseling, and health management initiatives offer potential avenues to alleviate loneliness among the elderly, thereby enhancing their quality of life and well-being. In conclusion, this study provides valuable insights into the predictors and mitigating factors of loneliness among elderly individuals in China, underscoring the multifaceted nature of loneliness and the potential of machine learning in understanding and addressing this critical issue in aging populations.

## 5. Conclusion

This study successfully developed a machine learning-based prediction model for loneliness among elderly individuals by identifying and analyzing 16 predictive factors. After comprehensive comparison and considering factors such as economic impact and ease of implementation, logistic regression was chosen as the model, although this does not imply that a multilayer perceptron model is necessarily inferior. The feature importance ranking revealed that cognitive function, specifically the MMSE score, is the most critical predictor of loneliness, with higher MMSE scores being associated with lower occurrences of loneliness. The risk prediction model demonstrated high performance and accuracy, surpassing traditional statistical methods, and provides a scientific basis for early intervention in elderly loneliness. Despite the significant results, the study has limitations, including the sample primarily being from China, which necessitates further validation of the model’s applicability in different contexts. Additionally, limitations inherent to the researchers, such as constraints in expertise, prevented optimization of the model parameters to enhance accuracy. Future research should incorporate additional biomarkers and behavioral data to further optimize and improve the model’s generalizability. Moreover, personalized intervention strategies for high-risk elderly populations need further exploration to effectively reduce loneliness and enhance quality of life and well-being. Overall, this study offers new insights into predicting elderly loneliness and lays the groundwork for targeted interventions, advancing the health and welfare of the elderly population. It provides crucial scientific support for public health policy development and helps address the challenges posed by global population aging.

## Figures and Tables

**Figure 1:**
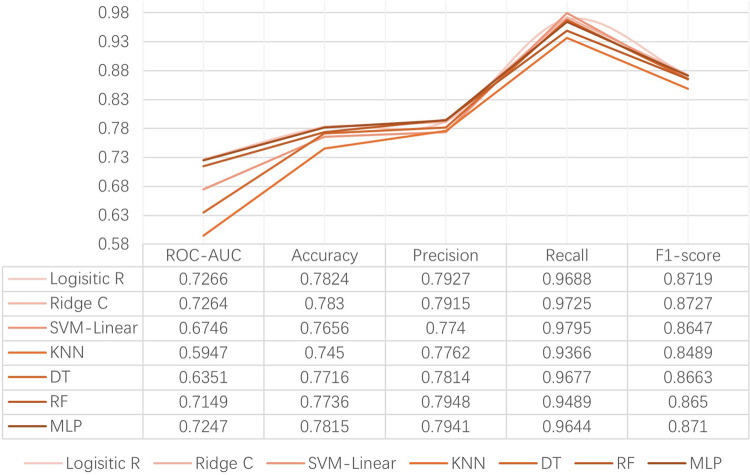
Evaluation Metrics for Seven Risk Prediction Models Based on Machine Learning

**Table 1:** Summary of Variable Feature Selection: Integration of Univariate and Multivariate Analysis. This table consolidates the findings from both univariate and multivariate analyses to highlight key variable features for predicting loneliness among elderly individuals.

Variables	ANOVA					Multiple Regression Analysis				
	β	S.E	Z	*P*	OR (95%CI)	β	S.E	Z	*P*	OR (95%CI)
Trueage	0.002	0.002	1.523	0.128	1.002 (0.999 ~ 1.006)					
Years Of Schooling	−0.032	0.005	−6.434	**<.001**	0.968 (0.959 ~ 0.978)	−0.007	0.006	−1.100	0.271	0.993 (0.982 ~ 1.005)
Katz	0.045	0.011	4.040	**<.001**	1.046 (1.023 ~ 1.069)	0.059	0.015	3.876	**<.001**	1.061 (1.030 ~ 1.093)
IADL	−0.029	0.006	−5.124	**<.001**	0.971 (0.961 ~ 0.982)	−0.035	0.010	−3.577	**<.001**	0.966 (0.948 ~ 0.984)
Number Of Chronic	0.080	0.011	7.322	**<.001**	1.083 (1.060 ~ 1.107)	0.056	0.013	4.224	**<.001**	1.057 (1.030 ~ 1.085)
gender										
female					1.000 (Reference)					1.000 (Reference)
male	−0.198	0.038	−5.194	**<.001**	0.820 (0.761 ~ 0.884)	0.064	0.047	1.373	0.170	1.066 (0.973 ~ 1.168)
**Residence**										
Urban					1.000 (Reference)					1.000 (Reference)
Rural	0.191	0.046	4.110	**<.001**	1.210 (1.105 ~ 1.325)	0.099	0.058	1.708	0.088	1.104 (0.986 ~ 1.236)
Co-residence										
Family members					1.000 (Reference)					1.000 (Reference)
Alone	1.002	0.045	22.405	**<.001**	2.725 (2.496 ~ 2.975)	0.735	0.052	14.169	**< .001**	2.085 (1.883 ~ 2.308)
In a institution	0.589	0.094	6.289	**<.001**	1.801 (1.499 ~ 2.164)	0.584	0.113	5.189	**< .001**	1.793 (1.438 ~ 2.236)

no					1.000 (Reference)					1.000 (Reference)
yes	0.514	0.051	10.101	**< .001**	1.673 (1.514 ~ 1.848)	0.297	0.057	5.198	**< .001**	1.346 (1.203 ~ 1.506)
Purifiers										
no					1.000 (Reference)					1.000 (Reference)
yes	−0.158	0.072	−2.195	**0.028**	0.854 (0.741 ~ 0.983)	0.000	0.078	0.004	0.997	1.000 (0.859 ~ 1.165)
Quality Of Life										
bad					1.000 (Reference)					1.000 (Reference)
good	−1.430	0.094	−15.180	**< .001**	0.239 (0.199 ~ 0.288)	−0.672	0.110	−6.100	**< .001**	0.511 (0.412 ~ 0.634)
Health										
bad					1.000 (Reference)					1.000 (Reference)
good	−0.995	0.050	−20.071	**< .001**	0.370 (0.335 ~ 0.407)	−0.693	0.059	−11.713	**< .001**	0.500 (0.446 ~ 0.562)
Personality										
introvert					1.000 (Reference)					1.000 (Reference)
extrovert	−1.185	0.086	−13.771	**< .001**	0.306 (0.258 ~ 0.362)	−0.771	0.097	−7.928	**< .001**	0.462 (0.382 ~ 0.560)
Smoke										
no					1.000 (Reference)					
yes	−0.106	0.054	−1.953	0.051	0.899 (0.808 ~ 1.000)					

no					1.000 (Reference)					1.000 (Reference)
yes	−0.333	0.058	−5.699	**<.001**	0.717 (0.639 ~ 0.804)	−0.245	0.065	−3.752	**<.001**	0.783 (0.689 ~ 0.890)
Exercise										
no					1.000 (Reference)					1.000 (Reference)
yes	−0.342	0.043	−7.937	**<.001**	0.710 (0.653 ~ 0.773)	−0.312	0.050	−6.193	**<.001**	0.732 (0.663 ~ 0.808)
Interact With Friends										
no					1.000 (Reference)					1.000 (Reference)
yes	0.139	0.038	3.680	**<.001**	1.149 (1.067 ~ 1.238)	0.179	0.048	3.734	**< .001**	1.196 (1.089 ~ 1.313)
Satisfaction Of Finance										
Not enough					1.000 (Reference)					1.000 (Reference)
Enough	−0.656	0.049	−13.394	**<.001**	0.519 (0.471 ~ 0.571)	−0.432	0.056	−7.710	**< .001**	0.649 (0.582 ~ 0.725)
Marital Status										
Currently married and living with spous					1.000 (Reference)					1.000 (Reference)
Separated	0.476	0.149	3.189	0.001	1.610 (1.202 ~ 2.158)	0.293	0.160	1.835	0.067	1.340 (0.980 ~ 1.832)
Divorced	1.347	0.285	4.727	**<.001**	3.845 (2.200 ~ 6.720)	0.749	0.333	2.249	**0.025**	2.115 (1.101 ~ 4.064)
Widowed	0.819	0.043	19.269	**< .001**	2.269 (2.088 ~ 2.467)	0.843	0.054	15.599	**< .001**	2.323 (2.089 ~ 2.582)
Never married	1.679	0.173	9.714	**< .001**	5.360 (3.820 ~ 7.522)	1.303	0.193	6.740	**< .001**	3.679 (2.519 ~ 5.373)
Visual Function										
Not impaired function					1.000 (Reference)					1.000 (Reference)
Impaired function	−0.330	0.038	−8.593	**< .001**	0.719 (0.667 ~ 0.775)	−0.403	0.047	−8.603	**< .001**	0.668 (0.610 ~ 0.732)
Hearing										
Not impaired function					1.000 (Reference)					1.000 (Reference)
Impaired function	0.089	0.038	2.343	**0.019**	1.093 (1.015 ~ 1.178)	0.036	0.047	0.775	0.439	1.037 (0.946 ~ 1.137)
Toothache										
no					1.000 (Reference)					1.000 (Reference)
yes	0.329	0.050	6.588	**< .001**	1.390 (1.260 ~ 1.533)	0.153	0.065	2.368	**0.018**	1.165 (1.027 ~ 1.323)
Pain In Jaw										
no					1.000 (Reference)					1.000 (Reference)
yes	0.475	0.065	7.306	**< .001**	1.608 (1.415 ~ 1.826)	0.158	0.083	1.905	0.057	1.172 (0.995 ~ 1.379)
Fallen History										
no					1.000 (Reference)					1.000 (Reference)
yes	0.272	0.044	6.213	**< .001**	1.312 (1.204 ~ 1.429)	0.123	0.049	2.511	**0.012**	1.131 (1.027 ~ 1.245)
Calcium										
no					1.000 (Reference)					1.000 (Reference)
yes	−0.190	0.073	−2.606	**0.009**	0.827 (0.716 ~ 0.954)	−0.261	0.080	−3.267	**0.001**	0.770 (0.659 ~ 0.901)

OR: Odds Ratio, CI: Confidence Interval

**Table 2:** Homogeneity Test of Training and Validation Sets

Variable	Train_Mean	Train_SD	Test_Mean	Test_SD	P_Value
MMSE	0.77694902	0.28934831	0.76892037	0.29406356	0.11721721
katz	0.10858127	0.03604885	0.10884847	0.03625919	0.67334883
IADL	0.0716741	0.05226146	0.070834	0.05090466	0.34983853
number_of_chroic	0.05963166	0.06851699	0.05847106	0.06535877	0.31763571
coresidence	0.87149094	0.87149094	0.87149094	0.87149094	0.87149094
smell	0.6475347	0.6475347	0.6475347	0.6475347	0.6475347
quality_of_life	0.36689445	0.36689445	0.36689445	0.36689445	0.36689445
health	0.35229393	0.35229393	0.35229393	0.35229393	0.35229393
personality	0.84178341	0.84178341	0.84178341	0.84178341	0.84178341
drink	0.94691598	0.94691598	0.94691598	0.94691598	0.94691598
exercise	0.46505425	0.46505425	0.46505425	0.46505425	0.46505425
interact_with_friends	0.58930351	0.58930351	0.58930351	0.58930351	0.58930351
satisfaction_of_finance	0.75175596	0.75175596	0.75175596	0.75175596	0.75175596
marital_status	0.06017544	0.06017544	0.06017544	0.06017544	0.06017544
visual_function	0.11983043	0.11983043	0.11983043	0.11983043	0.11983043
calcium	0.59701035	0.59701035	0.59701035	0.59701035	0.59701035

## Data Availability

The data used in this study were from the “China Aging Health influencing Factors Follow-up Survey” organized and managed by the Research Center of Healthy Aging and Development of Peking University; The survey was jointly funded by the National Natural Science Foundation of China (project approval numbers 71233001 and 71110107025), NIH(Project approval numbers R01AG023627), and the United Nations Population Fund.
